# Profil clinique et évolutif des lésions de la peau et des parties molles chez les diabétiques en 2017 à la salle de pansement du Centre Marc Sankale de Dakar

**DOI:** 10.11604/pamj.2019.32.209.18524

**Published:** 2019-04-29

**Authors:** Diallo Ibrahima Mané, Diédhiou Demba, Sow Djiby, Ndour Michel Assane, Barrage Ahmet Limane, Ka-Cissé Marie, Sarr Anna, Ndour Mbaye Maimouna

**Affiliations:** 1 Clinique Médicale II, Centre Hospitalier Abass Ndao, UCAD, Dakar, Sénégal

**Keywords:** Peau et parties molles, diabète, Dakar, Skin and soft tissues, diabetes, Dakar

## Abstract

**Introduction:**

Le but de notre étude était de déterminer le profil clinique et évolutif des lésions de la peau et des parties molles des sujets diabétiques suivis à la salle de pansement.

**Méthodes:**

Il s'agissait d'une étude observationnelle descriptive et analytique menée du 1^er^ janvier au 31 décembre 2017 à la salle de pansement du centre Marc Sankale de Dakar. Notre étude a porté sur les sujets diabétiques ayant consultés à la salle de pansement.

**Résultats:**

Au total, 37173 actes de soins ont été enregistrés au centre Marc Sankale. Les activés de soins à la salle de pansement représentaient 16418 cas soit une prévalence de 14,16%. L'âge moyen était de 56,6 ± 12 ans et le sex ratio (H/F) de 0,88. Le diabète de type 2 prédominait (78,97%) et la durée moyenne du diabète était de 8,06 ± 7,9 ans. La glycémie capillaire moyenne était de 2,4 ± 1 g/l. La neuropathie diabétique était présente chez 72,33% des cas. Les lésions se situait aux membres dans 93,98% (1185 cas). Les lésions les plus représentatives étaient l'ulcère (46,76%), l'abcès (13,46%), le phlegmon (13,20%), la gangrène (8,41%), l'érysipèle (3,78%), le mal perforant (3,53%), l'intertrigo (3,95%). Les lésions étaient infectieuses (61,41), non infectieuses (33,50%), vasculaires pures (1,57%) et Mixtes (3,70%). Sur les 1189 patients 7,57% avaient présentés une ostéite. Les germes retrouvés étaient des bactéries grams positifs (12,70%), grams négatifs (23,80%). L'amputation était corrélée à la topographie de la lésion (p=0.00), au type de lésion (p=0.000), à l'ancienneté du diabète (p=0,02), au type de diabète (p=0,008), à la présence d'ostéite (p=0,006). L'amputation etait mineur (43,33%), et majeur (37,43%). Nous avons enregistré 70 décès (5,89%).

**Conclusion:**

Les lésions de la peau et des tissus mous restent dominées par le pied diabétique. La mortalité est non négligeable et le risque d'amputation était statistiquement corrélé à la topographie, au type de lésion, à l'ancienneté et le type de diabète et à l'existence d'ostéite.

## Abstract (en)

**Introduction:**

This study aims to determine the clinical and evolutionary profile of skin and soft tissue lesions in diabetics followed up at the dressing room.

**Methods:**

We conducted an observational descriptive and analytical study in the dressing room of the Marc Sankalé Diabetes Center, Dakar from 1 January to 31 December 2017. Our study focused on diabetics who were examined in the dressing room.

**Results:**

A total of 37.173 procedures were recorded at the Marc Sankalé Diabetes Center; 16.418 patients were treated in the dressing room, representing a prevalence of 14.16%. The average age of patients was 56,6 ±12 years, the sex ratio (M/F) was 0,88. Type 2 diabetes predominated (78,97%) and the average disease duration was 8.06 ± 7.9 years. The mean capillary blood glucose concentration was 2.4±1 g/l. Diabetic neuropathy was found in 72.33% of patients. Limb lesions were found in 93,98% of patients(1185 cases). The most common lesions included: ulcer (46,76%), abscess (13.46%), phlegmon (13.20%), gangrene (8.41%), erysipelas (3.78%), perforating ulcer (3.53%), intertrigo (3.95%). The lesions were be divided into infectious lesions (61,41), non-infectious lesions(33,50%), pure vascular lesions (1.57%) and mixed lesions (3.70%). Out of 1189 patients 7.57% had had osteitis. The germs found were Gram-positive bacteria (12.70%), Gram-negative bacteria (23.80%). Amputation was performed according to the topographical features of the lesion (p=0.00), the type of lesion (p=0.000), the seniority of diabetes (p=0.02), the type of diabetes (p=0.008), the presence of osteitis (p=0.006). Minor amputation was performed in 43,33% of cases, major amputation in 37.43% of cases; 70 deaths were recorded (5.89%).

**Conclusion:**

Skin and soft tissue lesions were dominated by diabetic foot. Mortality is not negligible and the risk of amputation was statistically related to the topographical features of the lesion, the type of lesion, the seniority and the type of diabetes and the presence of osteitis.

## Introduction

La prévalence du diabète dans le monde est de 8,8% en 2017 et 79% vivent dans des pays à faible et moyen revenu. L'hyperglycémie est le primo movens des complications micro et macro vasculaire [1]. Dans le contexte africain, l'infection en raison d'une altération de fonction de phagocytose et de chimiotactisme des défenses immunitaires [2, 3], est souvent le facteur révélateur du diabète [4]. Au Sénégal, l'enquête STEP de 2015 révèle une prévalence du diabète à 2,1% [5]. En raison des complications micro et macro vasculaires qu'engendrent cette endémie, la mortalité infectieuse non négligeable. Dans la série de Diedhiou *et al*. [6]. Chez les patients hospitalisé, la mortalité était de 15,7%, dont 54,8% décès pour une pathologie infectieuse. Le centre anti diabétique Marc Sankale est une référence nationale dans la prise en charge du diabète. La plupart des lésions de la peau et des parties molles y sont prise en charge. La salle de pansement est devenue en moins de 10 ans une référence et une victime de sa réputation au vu de la pléthore de patient qui y sont suivis. Les quelques données parcellaires sur le pied et la main rapportaient des résultats diverses et variés. Les lésions du pied (2%) des consultations de diabétologie, sont responsables de 28% des amputations avec une mortalité de 15% [7]. La localisation à la main (9,1%) avaient une mortalité y représentait 4,2% des cas [8]. La problématique actuelle est que les lésions de la peau et des parties molles au centre Marc Sankale constitue un problème de santé publique à l'image du diabète. La connaissance du profil clinique et évolutif des patient qui y sont suivis et des facteurs liés à l'amputation pourrait aider à apprécier l'activité de la salle de pansement et à améliorer la prise en charge des patients. L'objectif était de déterminer le profil des lésions rencontrées, leurs devenirs et les facteurs lies à l'amputation des diabétiques suivis en salle de pansement.

## Méthodes

Il s'agissait d'une étude observationnelle descriptive et analytique menée du 1^er^ janvier 2017 au 31 décembre 2017, réalisée à la salle de pansement du centre Marc Sankale. Tous les dossiers des patients diabétiques ont été inclus. Les patients sans dossier et les dossiers sans numéros de téléphone étaient exclus de l'étude. L'âge était reparti en intervalle suivant le rapport de l'agence national de la démographie et de la statistique du Sénégal: enfants < 15 ans, adultes de 15 et 64 ans et sujet âgé > 65 ans. La glycémie du jour, de type capillaire, était effectuée à partir des glucomètres marque Accu Check active. L'hypoglycémie, la normoglycémie et l'hyperglycémie était définie suivant les critères de l'American Diabètes Association [9]. Le mécanisme lésionnel traumatique était retenu pour les patients qui ont relatés un contexte traumatique. Le siège de la lésion sur les parties du corps suit une répartition classique (tête, tronc et 4 membres). Le diagnostic de la lésion était présomptif base sur les éléments cliniques et paracliniques. La neuropathie était retenue devant les signes cliniques et le test au mono filament 10 mg de Semmes-Weinstein. Le test au diapason gradué de Riedle-Seiffer n'a pas était réalisé en raison de son indisponibilité. La recherche des réflexes ostéo-tendineux était systématique pour les atteintes des membres. L'artériopathie était diagnostiquée grâce à la classification de Leriche et fontaine, par la palpation des pouls périphériques, l'aspect caractéristique du pied [10]. En cas de diminution ou d'abolition, ou lorsque le contexte est évocateur, un doppler artériel était demandé. Un stylet métallique Hartmann permettait de rechercher le contact osseux en cas de lésion profonde, mais aussi d'apprécier la profondeur de certaine lésion. En cas de lésion profonde, une imagerie (radiographie) était systématiquement demandée pour rechercher une ostéite. Elle était à large spectre selon le profil de la lésion. Le prélèvement bactériologique se faisait après nettoyage et rinçage de la lésion avec une solution antiseptique à PH neutre et du sérum salé isotonique. Il s'intéressait soit au pus ou aux débris de chaire infecté prélevés en profondeur. L'antibiothérapie était initialement probabiliste puis guidée par la bactériologie en cas d'infection. La prescription d'antalgique était justifiée que par la présence d'une douleur. Apres évaluation EVA [11], les antalgiques de palier II étaient les plus utilisé seuls, ou en association avec le paracétamol. Un sérum antitétanique (SAT) était systématique chez tous les patients consultants à la salle de pansement. Les patients relevant d'une hospitalisation étaient envoyés en service de médecine interne de l'hôpital, ou en service de chirurgie pour les cas nécessitant un geste chirurgical urgent. Un appel téléphonique a permis d'évaluer le devenir des patients (séquelles, décès, guérison complète). Ceux qui n'étaient pas joignable après 3 tentatives à des jours différents, ou qui n'avait pas fait signe de vie, était considérés comme des perdus de vus. Tous ces éléments nous avaient permis de recueillir les paramètres épidémiologiques (âge sexe), cliniques, et évolutifs. La saisie et l'analyse des données ont été effectuées avec le logiciel EPI info. Le traitement de texte et la confection des tableaux ont été effectués avec les logiciels Word 2013 et Excel 2013. Un p value <0,05 était considérait comme significatif.

## Résultats

**Aspects sociodemographiques:** Au total, 37173 actes de soins ont été réalisés dans le centre Marc Sankale sur la période de l'étude. Les actes de soins de la salle de pansement représentaient 16418; soit une prévalence de 44,16%. Le nombre de nouveaux actes en salle de pansement était de 2366; soit une incidence de 6,36% pour tout le centre Marc Sankale et de 14,41% pour seulement la salle de pansement. L'âge moyen était de 56,6 ans ±12 ans, avec un sexe ratio de 0,88. Selon la tranche d'âge, il s'agissait de 0,17%, 24,4% et 75,4% des lésions respectivement chez les enfants, l'adulte et le sujet âgé. La plupart des patients (87,1%) étaient venu de leur propre chef.

**Étude du diabète:** Le diabète de type 2 prédominait dans 78,97% des cas. Chez 1,27% des patients le type de diabète n'a pas était précisé. La durée moyenne du diabète était de 8,06 ± 7,9 ans avec une médiane de 23 ans. L'ancienneté du diabète était inferieur à 5 ans dans 53,11% et de plus de 10 ans dans 41,80%. Le diabète était inaugural dans 10,18% des cas. La glycémie capillaire moyenne était de 2,4 ± 1 g/l pour une médiane de 2,35 g/l. Des hypoglycémies (0,78%) ont été retrouvées dans notre étude. Une hyperglycémie majeure supérieure à 3 g/l représentait 27,95%. La neuropathie était présente chez 72,33%. La présence d'une neuropathie était plus visible entre la 1^ère^ et la 5^ème^ année de diabète et chez les patients ayant plus de 10 ans d'évolutivité du diabète. L'artériopathie était présent chez 49,77% sur les 227 (17,9%) patients ayant réalisé un échographie Doppler. Les principaux facteurs de risque et/ou complications associées au diabète étaient une hypertension artérielle (14,81%), une néphropathie (0,95%) et une rétinopathie (0,39%) dans le reste des cas les patients n'ont pas relaté de pathologies associées.

**Figure 1:**
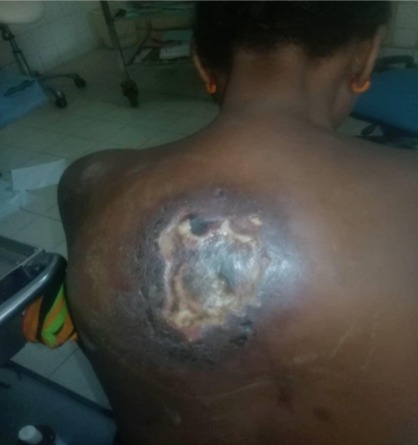
Abces necrotique du dos

**Figure 2:**
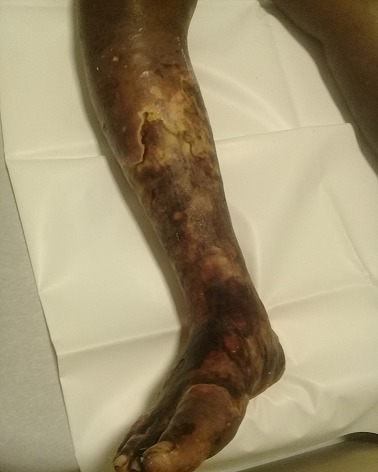
Gangrene mixte de la jambe et du pied droit

**Figure 3:**
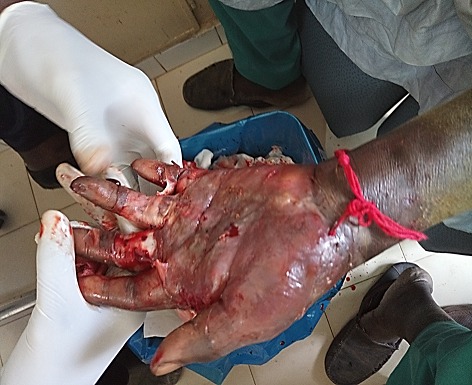
Phlegmon de la main gauche

**Etude de la lésion:** Le délai moyen de consultation était de 10,7±35 semaines, une médiane de 2 semaines. Les patients qui avaient consulté dans le mois d'apparition de la lésion représentaient 74,40% des cas (939 patients). Les lésions étaient d'apparition spontanée, chez 802 (63,54%). La localisation de la lésion se situait aux membres dans 93,98% (1185 cas), suivi du tronc (4,75%) et la tête (1,34%) (Figure 1, Figure 2, Figure 3). Les lésions étaient infectieuses dans 61,41% (775 cas), vasculaires pures dans 1,57% (20 cas) et mixtes dans 3,70% (44 cas). Les lésions les plus représentatives étaient l'ulcère (46,76%), l'abcès (13,46%), le phlegmon (13,20%), la gangrène (8,41%), l'érysipèle (3,78%), le mal perforant (3,53%), l'intertrigo (3,95%). Les 3,04% des lésions restantes étaient constituées par les bartholinites, les furoncles, les escarres, les fissures, les fasceites, les hématomes, les myosites, les ongles incarnés, l'onyxis (Tableau 1). L'ostéite représentait 7,57% de la population d'étude. Parmi les lésions infectieuses seul 40,65% ont bénéficié d'un prélèvement bactériologique. Il s'agissait de germes grams positifs (12,70%), grams négatifs (23,80%). Dans 2,23% des cas, la flore était poly microbienne et dans 61,23% on n'avait pas retrouvé de germe. Le Tableau 2 montre le profil des germes retrouvés (Tableau 2).

**Tableau 1:** Répartition des patients selon le type de lésions de la peau et des parties molles

LESIONS	NOMBRE (1262)	PREVALENCE
Intertrigo	50	3,95%
Mal perforant	44	3,53%
Ulcère	590	46,76%
Abcès	170	13,46%
Panaris	49	3,87%
Phlegmon	166	13,20%
Erysipèle	48	3,78%
Gangrène	106	8,41%
Autres	39	3,04%

**Tableau 2:** Répartition des patients selon le germe isolé

GERMES	NOMBRE (315)	PREVALENCE (%)
Staphylococcus aureus	24	7,61
Streptococcus entérococcus	16	5,07
Proteus mirabilis	6	1,90
Escherichia Coli	19	6,02
Flore poly microbien	7	2,22
Pseudomonas	50	15,87
Pas de germe	193	61,23

Presque tous les patients ont reçu un traitement antibiotique. Il s'agissait de: une mono antibiothérapie chez 155 patients (13,59%) avec comme chef de file l'oxacilline; une bi-antibiothérapie dans 46,59% des cas. Les associations les plus utilisées étaient amoxicilline + acide clavulanique (37,51%) et les quinolones associées aux imidazoles (3,36%); la tri-antibiothérapie et la quadri-antibiothérapie était respectivement utilisé dans 15,31% et 31,37%; seul 8,40% des patients n'avaient pas de traitement antibiotique; Un traitement anti mycosique était mis en place dans 27% des cas. L'acte chirurgical était significativement corrélé à la topographie de la lésion (membre inferieur (OR=3,18(1,46-6,94), p=0,00), membre supérieur (OR=0,21 (0,05-0,59), p=0.00)), le type de lésion (abcès (OR=0,20 (0,06-0,51), p=0.000), gangrène (OR=12,98(8,19-20,61), p=0.000)), à l'ancienneté du diabète (OR=0,64 (0,4-1,00) p=0,02), au type de diabète (OR=0,48 (0,24-0,89) p=0,008), la présence d'ostéite (OR=0,42 (0,21-0,83) p=0,006). Le geste chirurgical le plus retrouvé était l'amputation du membre inferieur avec au-devant l'amputation mineur (orteil (43,33%)), amputation majeur (jambe (28,33%), cuisse (6,60%) et l'avant pied (2,50%)) et un débridement (15%). En ce qui concernait le membre supérieur seul des amputations de doigt était retrouvé (4,16%). Le décès était retrouvé chez 46 (7,82%) des cas, la guérison chez 71,09% des patients, et seul 124 (21,09%) avait toujours leur pansement en cours. Parmi les patients décédés l'atteinte du membre supérieur représentaient 5,72% (4 cas) le membre inferieur (63 cas) 88,58%.

## Discussion

**Limites de l'étude:** Les limites rencontrées étaient principalement: certains dossiers incomplets par rapport aux explorations pour des raisons d'accessibilités financière et géographique; la profession des patients n'était pas disponible; les difficultés de suivi à long terme par le canal téléphonique.

**Aspect socio-démographiques:** Dans notre étude la prévalence des lésions de la peau et des parties molles au centre Marc Sankale était de 44%. Cette prévalence semble indiqué les activités du centre et justifie son nom centre de référence. Elle était inférieure à celle retrouvée dans les données de la littérature où des fréquences de 51,1 à 97% étaient rapportées [12]. La population appartenait presque à la 5^ème^ décade de vie avec une prédominance féminine. Cette moyenne d'âge était similaire à la plupart des études. Cependant, une prédominance masculine était relatée dans des études africaines [12-16] et espagnole [17]. Parmi les morbidités associées à ces lésions de la peau et parties molles, les pathologies cardiovasculaires (15%) prédominaient. Ce qui était le cas pour une série indienne [18] où les affections cardiovasculaires dominées dans les lésions cutanées et sous cutanées. Les mêmes constats ont été faits par Mahajan *et al*. [19] chez les sujets hypertendus diabétiques. Ceci suggère que les pathologies cardiovasculaires doivent être considérées comme un éventuel facteur favorisant de la survenue des lésions de la peau et des parties molles chez les diabétiques. Notre étude nous montre que les lésions de la peau et des parties molles prédominent chez les diabétiques anciens porteurs de pathologies cardiovasculaires.

**Etude du diabète:** Les complications cutanées sont souvent décrites comme inhérent à un diabète ancien. Dans notre étude, l'ancienneté du diabète était en moyenne de 8,06 ± 7,9 années, avec une prédominance du diabète de type 2. Ceci a été rapporté par beaucoup d'auteurs. Cette moyenne est très voisine de celle observée par Foss au Brésil [20]. Au Sénégal, Diédhiou *et al*. [21], Ndour *et al*. [8] sur des études portantes respectivement sur les affections du pied et de la main retrouvaient une ancienneté du diabète similaire à notre étude avec une prédominance du type 2. D'autres auteurs avaient fait le même constat [22, 23]. Les troubles cutanés chez les patients atteints de diabète sont fortement corrélés au contrôle glycémique. À titre d'exemple, Foos *et al*. [20] avaient mené une étude auprès de 403 patients atteints de diabète au Brésil. L'étude avait montré que 94% des patients dont le contrôle glycémique était insuffisant présentaient un trouble de la peau d'une part. D'autre part, seul 60% des patients atteints de diabète sucré avec un contrôle adéquat de la glycémie avaient une maladie de la peau. Dans notre série la glycémie moyenne de nos patients était de 2,35 g/l. Plus de 60% des patients avaient une glycémie capillaire supérieure à 2 g/l. Dans la plupart des séries africaines touchant les lésions de la peau et les parties molles à travers le pied ou la main, on retrouvait un diabète déséquilibre avec des glycémies moyenne supérieur à 2g/l [8, 21, 22]. Ce phénomène d'hyperglycémie induit la formation d'agent de glycathion non enzymatique (AGE) qui, modifie les propriétés du collagène (diminue la flexibilité et la solubilité et augmente sa rigidité) participant ainsi à sa rigidité et au vieillissement de la peau qui devient fragile [24].

**Etude des lésions de la peau et des parties molles:** Dans notre étude plus de la moitié des patients avaient consultés dans le mois qui suit la lésion et les lésions infectieuses étaient au devant. Ce constat était identique au Pakistan [25] et en Inde [26] avec des fréquences respectives de 26% et 60%. En Afrique, la plupart des études s'intéressent au pied de façon spécifique ou la main chez le diabétique. La gravité du diabète est liée à l'apparition et au développement de complications chroniques, qui touchent de nombreux organes, parmi lesquelles la peau et les parties molles. Selon Efrat *et al*. [27], les complications cutanées du diabète font partie de ses complications les plus dévastatrices. Elles sont liées à une haute morbidité ainsi qu'à un certain taux de mortalité. En effet, l'évolution du diabète est habituellement grevée de complications cutanées souvent faciles à diagnostiquer. L'absence ou le retard de prise en charge peut provoquer un drame pour le patient: dissémination d'une infection, amputation d'un pied diabétique. Les lésions infectieuses de notre étude venaient au premier plan chez plus de 55% de nos patients. Ce chiffre était supérieur à celui observé par Ibrahima *et al*. [27] en 1986 soit 9,5%. Feleke *et al*. [28] en 2007, ont trouvé un résultat inférieur au nôtre soit 12,8%. Les chiffres élevés de notre étude s'expliquent par l'ampleur que commence à prendre cette pandémie, une meilleure connaissance des lésions et la création de centre de prise en charge des lésions diabetiques comme le pied diabétique (biais de concentration).

Les lésions les plus retrouvées son représentées par: l'ulcère (46,76%), l'abcès (13,46%), le phlegmon (13,20%), la gangrène (8,41%), l'érysipèle (3,78%), le mal perforant (3,53%), l'intertrigo (3,95%). Ndour Mbaye *et al*. [8] dans une étude de la main ayant collige 71 patients retrouve comme lésions les plus fréquents le phlegmon (34 cas), le panaris (20 cas), l'abcès (11 cas), l'onyxis et périonyxis (3 cas), la gangrène (2 cas). Diedhiou *et al*. [21] sur une étude du pied retrouve la gangrène infectieuse (17,1%) alors que le phlegmon et/ou abcès représentaient 9,4%. Ceci s'explique par le fait que les malades recrutés dans la série de Diedhiou *et al*. [21] étaient hospitalisés avec une lésion très avancée et sur un terrain sensible à l'infection (le pied). Le traitement anti infectieux était systématique et adapté en fonction de l'antibiogramme ou de l'évolution des lésions. Dans notre étude, les pratiques de la prise en chargent du diabète reflétaient bien les recommandations ou l'insulinothérapie occupe une place essentielle. Dans notre étude la mortalité des lésions de la peau et des parties molles était de 7,82% et intéressait plus les membres: inferieurs (4,96%), supérieurs (0,31%). Pour l'atteinte du membre supérieur, Ndour *et al* [8] dans une étude sur les infections de la main retrouve une mortalité plus élevé qui s'explique par le fait que les lésions infectieuses étaient plus grave sur une durée plus courte qui n'intéressait que la main. La mortalité pour le pied au Sénégal [6] et au Bénin [29] était supérieur à la mortalité de notre étude car les patients étaient hospitalisés avec un pronostic plus engage, mais voisine de celle retrouve au Congo [30].

## Conclusion

Les lésions de la peau et des parties molles constituent à l'image du diabète un problème de santé publique majeur surtout en zone tropicale. Elles restent dominées par les lésions du pied. Elles surviennent en général chez le patient âgé avec un diabète de type 2 ancien, porteur de tares vasculaires justifiant ainsi l'appellation « dermopathie diabétique ». Les lésions sont dominées par les causes infectieuses, et l'atteinte prédominante est représentée par le pied diabétique.

### Etat des connaissances actuelles sur le sujet

- Le pied diabétique est la principale lésion rencontrée;
- Fortement corrélé à un déséquilibre du diabète;
- Lésions infectieuses sont les plus rencontrées.

### Contribution de notre étude à la connaissance

- Le seuil glycémique de fragilisation cutané est de 2g/l;
- Conforte la forte association cardiovasculaire et justifie le terme de dermopathie diabétique;
- L'organisation des services de soins permettait des taux de guérison spectaculaire malgré le retard de prise en charge.

## Conflits d’intérêts

Les auteurs ne déclarent aucun conflit d'intérêts.
